# The *hns* Gene of *Escherichia coli* Is Transcriptionally Down-Regulated by (p)ppGpp

**DOI:** 10.3390/microorganisms8101558

**Published:** 2020-10-10

**Authors:** Anna Brandi, Mara Giangrossi, Attilio Fabbretti, Maurizio Falconi

**Affiliations:** School of Biosciences and Veterinary Medicine, University of Camerino, 62032 Camerino, Italy; anna.brandi@unicam.it (A.B.); mara.giangrossi@unicam.it (M.G.); attilio.fabbretti@unicam.it (A.F.)

**Keywords:** *hns*, guanosine penta-tetra-phosphate, stringent response, transcriptional regulation

## Abstract

Second messenger nucleotides, such as guanosine penta- or tetra-phosphate, commonly referred to as (p)ppGpp, are powerful signaling molecules, used by all bacteria to fine-tune cellular metabolism in response to nutrient availability. Indeed, under nutritional starvation, accumulation of (p)ppGpp reduces cell growth, inhibits stable RNAs synthesis, and selectively up- or down- regulates the expression of a large number of genes. Here, we show that the *E. coli* *hns* promoter responds to intracellular level of (p)ppGpp. *hns* encodes the DNA binding protein H-NS, one of the major components of bacterial nucleoid. Currently, H-NS is viewed as a global regulator of transcription in an environment-dependent mode. Combining results from *relA* (ppGpp synthetase) and s*poT* (ppGpp synthetase/hydrolase) null mutants with those from an inducible plasmid encoded RelA system, we have found that *hns* expression is inversely correlated with the intracellular concentration of (p)ppGpp, particularly in exponential phase of growth. Furthermore, we have reproduced in an in vitro system the observed in vivo (p)ppGpp-mediated transcriptional repression of *hns* promoter. Electrophoretic mobility shift assays clearly demonstrated that this unusual nucleotide negatively affects the stability of RNA polymerase-*hns* promoter complex. Hence, these findings demonstrate that the *hns* promoter is subjected to an RNA polymerase-mediated down-regulation by increased intracellular levels of (p)ppGpp.

## 1. Introduction

The signaling molecules guanosine-tetraphosphate (ppGpp) and guanosine-pentaphosphate (pppGpp), collectively named (p)ppGpp, are the main mediators of “stringent response,” a common phenomenon occurring when bacteria face environmental stresses [1,2,3]. Two enzymes are responsible for the production of (p)ppGpp, the major RelA synthase, and the SpoT degradase that displays both (p)ppGpp degradation and synthetic activity [4]. In particular, when bound to ribosome, the RelA enzyme senses through its autoinhibitory domain and is being activated by the entrance of uncharged tRNAs into the ribosomal A-site, a circumstance occurring during amino acid starvation [5]. Thus, nutrients limitation causes an intracellular accumulation of (p)ppGpp that dramatically alters bacterial physiology and metabolism [6]. Under this condition, indeed, growth rate is slowed, stable RNA (rRNA and tRNA) production is inhibited, and the high levels of modified nucleotide differentially up- or down-regulate the expression of a large number of genes. The stringent response has been investigated for half a century, and solid evidence for (p)ppGpp acting directly on RNA polymerase (RNAP) has been already reported [7,8]. However, only two recent studies have provided deeper insights into the mechanism of action exerted by (p)ppGpp in modulation of transcription initiation from specific promoters; (p)ppGpp was found to target the *E. coli* RNAP and two binding sites for this alarmone have been localized on the core enzyme [9,10]. In fact, the first site is at the interface of β’ and ω subunits of RNAP, while the second one originates from the contact point of the transcription factor DksA with RNAP. Recently, by means of high-throughput sequencing technologies (RNA-seq), a complete collection of *E. coli* promoters, controlled in vivo by (p)ppGpp, has been provided [11]. This study shows that 757 genes are up- or down-regulated (at least twofold) within five minutes of continuous production of (p)ppGpp through the conditional expression of a mutated RelA lacking its autoinhibitory domain. Even if Sanchez-Vazquez et al. [11] widened the list of (p)ppGpp-responding promoters (~75% were not previously identified), under the experimental conditions used, the *hns* gene encoding the Histone-like Nucleoid Structuring (H-NS) protein was not identified. Similarly, also Aberg et al. [12] did not find any (p)ppGpp-dependent changes of *hns* expression by a microarray analysis.

The H-NS protein has been extensively investigated in our laboratory, and its biochemical characterization and the regulation of this gene date back to the late 1980s [13,14,15]. Currently, H-NS, in addition to its well-established role in the organization of bacterial chromatin, is recognized as a “universal repressor” involved directly or indirectly in the regulation of numerous genes (~200), most of them linked to stress responses and bacterial pathogenesis [16,17,18,19]. The basis for H-NS function as a gene silencer resides in its preferential binding to AT-rich curved DNA sequences [20,21], often found upstream of *E*. *coli* promoters and in its ability to induce bending of noncurved DNA [22], thus altering DNA conformation/topology and/or competing with RNAP and other regulators [23,24,25,26]. Genome-wide functional analyses have mapped ~1000 sites bound by H-NS, accounting for the regulation of 5% of the entire *E. coli* genome [27], including DNA sequences of exogenous origin acquired by horizontal gene transfer [28,29].

In a previous study [30], we have found that the activation of *hns*, upon cell resumption from stationary phase, is partially abolished by high levels of (p)ppGpp. Here, we have provided a deeper investigation on the role of (p)ppGpp in the regulation of *hns*, showing that in vivo level of *hns* mRNA inversely correlates with the intracellular concentration of the modified nucleotide. Accordingly, we show that (p)ppGpp is able to inhibit *hns* promoter activity through in vitro transcription assay, suggesting a direct action of this molecule on the RNA polymerase. This finding is further supported by electrophoretic mobility shift assays (EMSA) that reveals a (p)ppGpp-mediated destabilization of RNAP-*hns* promoter complex.

## 2. Materials and Methods

### 2.1. Bacteria Strains

*E. coli* strains used in this study were: MRE600 (*F-,rna*) [31]; CF2676 (*argA*::Tn10, *relA*+) an N99 derivative (*F-, galK2, rpsL, lambda-)* [32,33]; CF1753 (*argA*::Tn10, *relA1*) an MG1655 derivative (K12 strain, *F-, lambda-, ilvG-, rfb-50, rph-1*) [33]; CF3042 (*zib563*::Tn10, *spot203*, *relA1*) an N99 derivative [33,34]; KT793 carrying IPTG inducible RelA protein from pKT31 or a truncated form from pKT40 [35]. *E. coli* cells were grown at 37 °C in Luria-Bertani (LB) broth supplemented with 0.5% glucose [36] or in “Phosphates-free” medium (100 mM Tris-HCl, pH 7.7, 0.5% peptone, 10 mM NH_4_Cl, 0.7 mM NaNO_3_, 1 mM Na_2_SO_4_, 0.5 mM MgSO_4_·7H_2_O, 0.05 mM MnCl_2_·4H_2_O).

### 2.2. DNA Manipulation and General Procedures

Total RNA purified by hot phenol extraction [37] was subjected to northern blot and the *hns* mRNA was detected by using a specific 5′-end-labeled oligonucleotide (5′-GCGCACGAAGAGTACGG-3′) or [^32^P]-labeled *hns* probes obtained from pPLc11 [38] and labeled by random primer reaction [36]. The radioactivity associated to *hns* mRNA, normalized for 16S rRNA, was quantified by Molecular Imager FX (Bio-Rad, Hercules, CA, USA). Thin layer chromatography was performed as previously described [39] and quantified by Molecular Imager FX (Bio-Rad).

### 2.3. In Vitro Transcription

In vitro transcription assays were programmed with pKK*400::cat,* a pKK232-8 derivative, carrying the *hns* promoter and its upstream regulatory region up to position −313 [15]. Multiple round transcription reactions were carried out at 37 °C in a total volume of 45 µL transcription buffer, essentially as previously described, [30] and mRNA synthesized in vitro was determined by northern analysis using a [32P]-labeled cat probe derived from pCM7 (Pharmacia, New Jersey, NJ, USA). Radioactivity was quantified by Molecular Imager FX (Bio-Rad).

### 2.4. Electrophoretic Mobility Shift Assays

The 221 bp DNA fragment carrying the *hns* promoter (from position −159 to position +62) was amplified by PCR using as template pKK600 [15] and the primers Gen405 (5′-TTCAGAATTTTAAGTGCTTCGCTCA-3′) and Gen406 (5′-GTGCTGCGAAATCATCGGTGTAAA-3′). The oligonucleotide Gen406 used in PCR was previously radiolabeled with T4 kinase and [γ-^32^P]-ATP [36]. Reaction mix contained the labeled DNA (~0.3 pmol) and purified RNA polymerase (7 pmol) in 200 µL of Binding Buffer (40 mM Hepes KOH pH 8, 50 mM KCl, 10 mM MgAcetate, 0.5 mM 1,4-Dithiothreitol DTT and 0.005% NP40). The reaction mix was divided into two tubes: the first one containing ribo-Adenosine 5’-triphosphate (ATP) (final conc. 50 µM) and the second one containing the same amount of ribo-ATP and ppGpp (final conc. 500 µM). The two tubes were incubated at 30 °C and aliquots (15 µL) were withdrawn in a time course experiment. Binding of RNA polymerase to the DNA probe was stopped by adding 5U of heparin and placing the samples in ice before loading on a native acrylamide gel (6%). Radioactivity associated to free and bound DNA was quantified by Molecular Imager FX (Bio-Rad).

The E. coli RNA polymerase was purified according to Gross et al. [40]. The amounts of all polymerase subunits (polypepdide chains β, β’, α, and ω) and σ factor in the preparation were determined by sodium dodecyl sulfate (SDS)-polyacrylamide gel electrophoresis and the RNA-synthesizing capacity was verified in an in vitro transcription assay (not shown).

## 3. Results

### 3.1. Low Levels of (p)ppGpp Stimulate hns Expression

**Figure 1:**
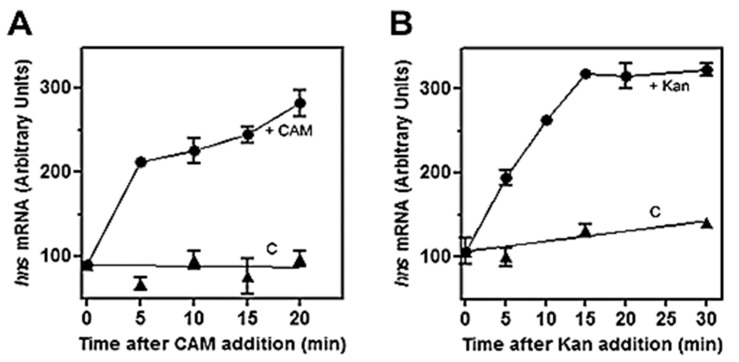
Effects of chloramphenicol and kanamycin on *hns* transcription in vivo. The *E. coli* strain MRE600 was grown at 37 °C in LB broth up to Abs_600_ = 0.5–0.6 (exponential phase of growth) and at the indicated times after the addition of 200 µg/mL of chloramphenicol (**A**) or 300 µg/mL of kanamycin (**B**), cell aliquots were harvested for RNA extraction. About 10 µg of total RNA were subjected to northern blot analysis and membranes hybridized with a [^32^P]-labeled *hns* probe. Treated cells (●) and control culture (C) in the absence of drugs (▲) are shown. Data represent the average of at least two independent experiments and standard deviation is reported.

**Figure 2:**
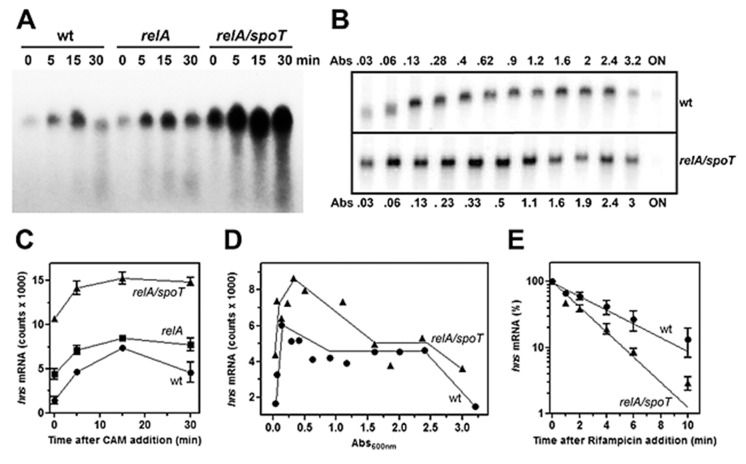
Chloramphenicol and mutations of *relA* and *spoT* genes induce *hns* transcription in vivo. The *E. coli* wt strain CF2676 (●) and its *relA* (■) and *relA/spoT* (▲) derivatives were grown at 37 °C in LB broth up to the exponential phase (Abs_600_ = 0.5–0.6). Then the steady-state level of *hns* mRNA was monitored by northern analysis at the indicated times after addition of chloramphenicol (200 µg/mL) (**A**) and as a function of the cell density (Abs_600_) (**B**). Lanes indicated with 0 in panels A, C, and E represent the *hns* mRNA levels in cells before drug addition, while ON in panel B is for Overnight Culture. Panels (**C**) and (**D**) show the quantifications of blots A and B, respectively. Determination of the chemical stability of *hns* mRNA in wt (●) and *relA/spoT* (▲) strains (**E**). Cultures at A_600__nm_ = 0.5 were treated with rifampicin (250 µg/mL) at time zero whose *hns* level is taken as 100%. After drug addition, RNA was extracted from cells harvested at the indicated times. The level of *hns* transcript was determined by northern blot hybridized with a [^32^P]-labeled *hns* probe. Data represent the average of at least two independent experiments and standard deviation is reported.

The role of (p)ppGpp on *hns* expression was initially investigated by means of antibiotics as chloramphenicol (CAM) and kanamycin (Kan), which were known to affect the intracellular level of this unusual nucleotide. In fact, these two drugs, by blocking protein synthesis, have been shown to cause a dramatic reduction in (p)ppGpp [41]. Thus, to verify whether (p)ppGpp could regulate *hns* expression, the level of *hns* mRNA was monitored in a time course experiment after treating the cell with high doses of CAM and Kan. As seen in Figure 1, both drugs behave similarly by triggering a prompt increase (~threefold) of *hns* transcript compared to the untreated cells as control. Steady-state level of *hns* mRNA was further investigated using a strain defective for RelA synthetase, the enzyme catalyzing (p)ppGpp production along with double mutant *relA/spoT* that, in addition to *relA,* carried an inactivated bifunctional (p)ppGpp synthetase/hydrolase SpoT. Xiao et al. [33] found that glucose exhaustion during cell growth provoked (p)ppGpp accumulation. Thus, the level of this effector progressively diminished passing from wild type (wt) to *relA* and from *relA* to *relA/spoT* backgrounds. Accordingly, northern analysis demonstrates that basal level of *hns* transcript (Figure 2A, time 0), shows ~ three- and sevenfold increase in *relA* and *relA/spoT* mutants, respectively, compared to wt strain. In addition, chloramphenicol treatment induces a sudden burst of *hns* expression in all the strains considered, even if at different extents with respect to the intrinsically different constitutive levels of *hns* mRNA (Figure 2A,C). Indeed, the lower the basal steady-state level, the greater is the induction of *hns* transcription caused by the CAM-dependent reduction in (p)ppGpp. Thus, this stimulation decreases from wt (fivefold) to *relA/spoT* (1.4-fold) through *relA* (twofold) cells; given that the intracellular level of (p)ppGpp is already very low in *relA/spoT* strain, CAM effects are almost completely lost in this double mutant (Figure 2C). To reinforce this finding, the level of *hns* transcript was measured during cell growth in wt and *relA/spoT* strains. Northern analysis reveals that *hns* expression is ~ twofold higher in double mutant compared to wt cells in the exponential phase of growth (A_600_ < 1) and this difference gradually disappears as the cells approach the stationary phase (A_600_ > 1.5) (Figure 2B,D). Taken together, these results strongly suggest that *hns* expression is inversely correlated with the intracellular concentration of (p)ppGpp.

Furthermore, to explore whether the higher amounts of *hns* transcript in cells carrying mutations in *relA* and *spotT* genes might simply reflect a stabilization of mRNA rather than an activation of *hns* promoter, the decay rate of *hns* mRNA was analyzed, comparing wt and *relA/spotT* double mutant after blocking the transcription by rifampicin (Figure 2E). The half-life (t_½_) of *hns* message, expressed as the time required for degrading 50% of the existing mRNA molecules, slightly changes in the two different genetic backgrounds, being t_½_ = 2.0 min and t_½_ = 1.2 min for wt and *relA/spoT* strains, respectively. Notably, the chemical stability of *hns* mRNA is higher in wt than in double mutant, ruling out that RNA t_½_ can account for the elevated levels of *hns* transcript observed in *relA/spoT* cells. Thus, these findings indicate that the *hns* promoter is responsive to (p)ppGpp, and the low level of this unusual nucleotide, as a consequence of the inactivation of *relA* and *spoT* genes, induces de novo transcription of *hns*.

### 3.2. High Levels of (p)ppGpp Directly Repress hns Promoter

**Figure 3:**
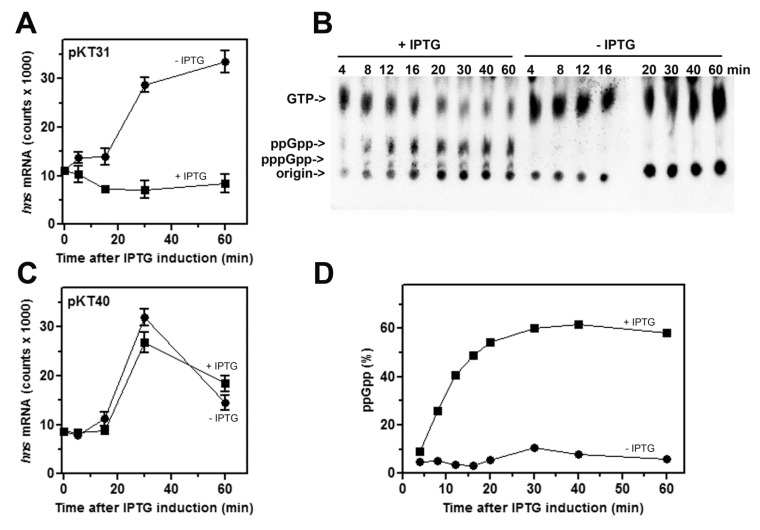
High levels of (p)ppGpp inhibit *hns* transcription in vivo. The level of *hns* transcript was monitored during growth in Phosphates-free medium at 37 °C in *E. coli* cells (KT793) expressing (**A**) or not (**C**) an active RelA synthetase. RNA to be subjected to northern blotting analysis was extracted from cells in stationary phase (time, 0; Abs_600 nm_ ≅ 3.0) and at the indicated times after a 10-fold dilution of cultures with fresh medium alone (●) or supplemented with 400 μM IPTG (■) to activate the *lacUV5::relA* gene. [^32^P]-labeled pppGpp, ppGpp and GTP, in IPTG-induced and not-induced pKT31 transformants, were resolved by thin layer chromatography (**B**) and radioactivity quantified by Molecular Imager. ppGpp, was expressed as percentage (%) of the total guanine nucleotides (**D**). The (p)ppGpp accumulation was not observed in IPTG-induced cells harboring plasmid pKT40 (not shown).

According to our previous observations [30], we tested the effect of high amounts of (p)ppGpp, linked to amino acid starvation, on the promoter activity of *hns*. To this aim, the intracellular (p)ppGpp levels were artificially increased by overexpressing the RelA alpha fragment in plasmid pKT31, carrying the (p)ppGpp catalytic domain independent of ribosome binding. The isogenic plasmid pKT40, because of the insertion of a stop codon in *relA* gene, encoded a truncated protein and was used as control. The production of functional RelA and its inactivated variant from pKT plasmids was achieved by adding IPTG to the cell cultures [35]. The nutritional upshift, as consequence of moving stationary-phase cells to a fresh medium not supplemented with IPTG, causes an abrupt burst (~threefold) of *hns* transcription (Figure 3A). Conversely, the accumulation of (p)ppGpp from pKT31 in induced cells, as determined by thin layer chromatography (Figure 3B,D), completely abolishes the transcriptional activity of *hns* promoter (Figure 3A). As control, the expression of a nonfunctional RelA enzyme from pKT40 does not affect the *hns* mRNA level regardless of IPTG induction (Figure 3C).

**Figure 4:**
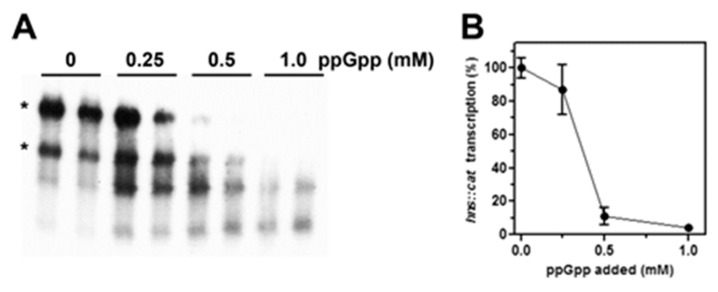
(p)ppGpp represses the in vitro transcription of *hns*. The activity of *hns* promoter was investigated by an in vitro assay as a function of increasing concentrations of (p)ppGpp. The supercoiled plasmid pKK400::*cat* (~100 ng) used as DNA template was incubated for 10 min at 37 °C with the *E. coli* RNA polymerase and the indicated amounts of (p)ppGpp. Then, transcription was stopped, and RNA analyzed by northern blot hybridized with a [^32^P]-labeled *cat* probe obtained from pCM7 (Pharmacia) (**A**). Asterisks indicate the two *cat* mRNAs that start from the *hns* promoter and end at the *rrnB* ribosomal RNA T1 or T2 terminators located downstream *cat* gene on pKK400. Transcription is expressed as percentage (%) taking the mRNA level in absence of (p)ppGpp as 100% (**B**). Reactions and processing of samples are described in detail in Materials and Methods. A representative gel is shown, and data correspond to the average of two independent experiments with duplicated points and standard deviation.

These results suggest that (p)ppGpp exerts a targeted repressive action on *hns* promoter, encouraging us to better investigate the effect of (p)ppGpp on *hns* transcription through an in vitro purified system as a function of increasing concentrations of this alarmone. As shown in Figure 4A,B, the addition of 0.5 mM of (p)ppGpp results in a residual *hns* transcription of ~10% and *hns::cat* mRNA becomes undetectable when this regulatory nucleotide is present at 1 mM, value that corresponds to the concentration estimated in vivo after amino acids starvation [42]. Importantly, suboptimal concentrations of (p)ppGpp (below 0.5 mM) that are not able to completely shut down the hns promoter activity, indeed causes the appearance of truncated cat transcripts (Figure 4A) as products of premature arrests of mRNA synthesis. This is possibly due to the pausing of RNA polymerase at transcriptionally unfavorable regions.

**Figure 5:**
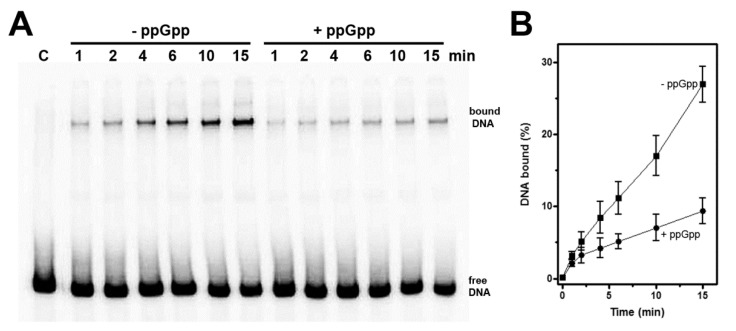
(p)ppGpp destabilizes the RNAP-*hns* promoter complex. EMSA was carried out as described in Materials and Methods by incubating for the indicated times, a [^32^P]-labeled DNA fragment (221 bp), corresponding to *hns* promoter with RNA polymerase in the absence or presence of ppGpp (500 µM) (**A**). The electrophoretic migrations of free DNA and *hns* DNA-RNAP complex are indicated and C is the control sample in absence of protein. Radioactivity associated to free and bound DNA was quantified and complexed DNA has been expressed as percentage (**B**). A representative gel is shown, and the average of four independent experiments and standard deviation are reported.

Ross et al. [9,10] demonstrated that (p)ppGpp was able to regulate transcription initiation from sensitive promoters by directly interacting with RNA polymerase. Thus, according to those findings and our in vitro transcription results (Figure 4), we explored the effects of (p)ppGpp on the interaction of RNAP with the promoter region of *hns*. The yield of DNA-RNAP complexes was investigated by electrophoretic mobility shift assay (EMSA) performed as a function of increasing periods of time without and with a fixed concentration of (p)ppGpp (Figure 5A). To convert unstable complexes into stable initiation complexes, the start ribonucleotide ATP was added. The RNAP-*hns* promoter binding kinetic was followed in a time course experiment and stopped using heparin, which, by irreversibly interacting with the free enzyme, prevented the aggregation of additional DNA-RNAP complexes [43]. As shown in Figure 5B, (p)ppGpp considerably slows down the formation of RNAP-*hns* promoter complex that is reflected in at least threefold reduction in the association constant (K_a_). Summarizing, EMSA results suggest that (p)ppGpp negatively affects the extent of complexes trapped in transcription that are therefore considered to be productive, hence accounting for transcriptional inhibition of *hns* promoter by (p)ppGpp as observed both in vivo and in vitro as well (Figure 3 and Figure 4).

## 4. Discussion

Bacterial cells, lacking homeostatic mechanisms, withstand changes in environmental conditions, deeply reshaping transcriptional activity. Hence, prompt adaptation to stresses, in particular to amino acid starvation, is coupled with an intracellular accumulation of the second messenger (p)ppGpp, also known as “magic spot”. This signaling molecule modulates the stringent response by affecting several fundamental cellular processes, including transcription.

In this study, we demonstrate that the expression of *hns* gene, coding for the DNA-binding protein H-NS, is under control of (p)ppGpp. Specifically, treatments with antibiotics capable of arresting the protein synthesis and null mutations in *relA* and *spoT* genes responsible for (p)ppGpp production reduce the alarmone level and as consequence elicit an activation of *hns* promoter in vivo (Figure 1 and Figure 2). Conversely, artificially increasing (p)ppGpp by a recombinant RelA-based system, causes a severe inhibition of the activity of *hns* promoter (Figure 3). According to these findings and since (p)ppGpp dramatically reprograms the entire transcription profile of bacterial cell, the fact that a regulatory gene as *hns* is a preferential target of this alarmone is not surprising, and may reflect a valid strategy to enhance the regulative ability of (p)ppGpp itself. In fact, H-NS, besides its role in DNA-packaging and nucleoid organization, shows high pleiotropic effects, and is currently considered as a global repressor of transcription in bacteria. The majority of genes belonging to H-NS regulon are involved in counteracting adverse growth conditions, and in particular H-NS is able to sense shifts of temperature, osmolarity, and pH [44]. Thus, (p)ppGpp, making H-NS decrease below a certain threshold level, might, in turn, determine a derepression of all the genes, commonly silenced by H-NS, which are essential to counteracting stress situation. This assumption is supported by the observation that other nucleoid-associated proteins, mostly those with pronounced regulatory functions such as FIS, Dps, DnaA, StpA, and Hfq, are selectively up- or down-regulated by (p)ppGpp [11]. In particular, FIS (Factor for Inversion Stimulation) was found to stimulate *hns* expression limited to the exponential phase of growth [45]. The lack of FIS in stationary phase cells might account for *hns* expression patterns of wt and *relA/spoT* strains that become similar as the cell density increases (Abs_600 nm_ > 1.5, Figure 2D). The fact is that (p)ppGpp represses both *fis* and *hns* promoters, thereby allowing us to speculate on the occurrence that the *hns* gene is subjected to a dual negative action by (p)ppGpp: (i) by directly impacting the intrinsic activity of *hns* promoter; (ii) by depleting the *hns* gene of its natural activator FIS.

Importantly, an adaptative stress-response also takes place when pathogenic bacteria face up transitions from free-living to host-associated states. In this contest, H-NS has been found in important human pathogens such as *S. typhimurium*, *S. flexneri*, *V. cholerae*, *Y. pesti*, *P. multocida* and, consistently, many genes controlled by this protein turn out to be virulence genes or genes expressing potential pathogenicity determinants [46,47,48,49,50,51]. The response of *hns* promoter to (p)ppGpp, investigated through an in vitro transcription system (Figure 4), undeniably indicates that the inhibition exerted by this modified nucleotide is direct and promoter specific. In fact, a 50% loss of promoter activity is observed at ~400 µM of (p)ppGpp and the *hns* transcription declines as much as 5–10% for higher (p)ppGpp concentrations. These amounts of (p)ppGpp are very close to those used in other in vitro studies [11,52,53] and consistent with the value (~0.9–1 mM) measured in vivo during the stringent response [42,54].

The failure to identify *hns* as a (p)ppGpp-responsive gene using transcriptomics techniques as RNA-seq [11] and microarrays [12] was quite unexpected. Possible explanations for conflicting results are reported below. As already stated, Sanchez-Vazquez et al. [11] estimated the *hns* mRNA level at 5 and 10 min after the production of (p)ppGpp by an inducible *relA* plasmid system. Using an analogous experimental approach (Figure 3A), we show that at least 20–25 min is required to detect significant differences in *hns* expression between induced and not-induced cells. Concerning the study by Aberg et al. [12], the total RNA for DNA chip and Reverse Transcriptase PCR experiments was extracted from cells in stationary phase (Abs_600_ = 1.5). Under our experimental conditions (Figure 2B,D), at cell density ranging from Abs_600_ = 1.5 to Abs_600_ = 3.0, the level of *hns* transcript of wt strain is comparable with that of *relA/spoT* double mutant, whereas a differential expression is observed mainly in exponential phase of growth. Thus, because of narrow time window or cell status, the in vivo (p)ppGpp-mediated regulation of *hns* might have been overlooked in previous studies [11,12]. Our results suggest that stationary phase cells slow down the *hns* transcription rate in a (p)ppGpp-independent manner probably because, at this time, other factors (i.e., the lack of FIS) may contribute mostly to the regulation of this gene. Remarkably, H-NS is also able to bind and repress transcription from its own promoter [15] and it was found to accumulate when cells enter the stationary phase [55]. However, the intracellular levels of this protein during growth-curve still remain a matter of debate [56,57,58]. Thus, an increase in H-NS level would enhance the autorepression providing an explanation for the decline of *hns* mRNA in both wt and *relA/spoT* strains, as observed at the onset of stationary phase (Abs_600_ = 1.5 in Figure 2D). Ultimately, stationary cells (as the starved ones), characterized by a low growth rate and a reduced DNA synthesis need a poor demand for de novo production of the nucleoid protein H-NS.

**Figure 6:**
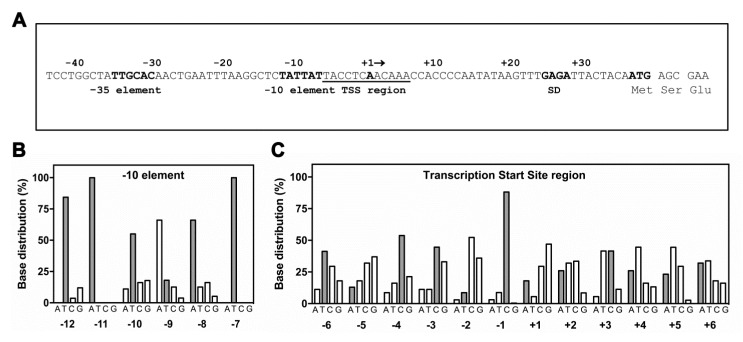
Matching of *hns* promoter sequence with base distributions for (p)ppGpp-inhibited promoters. The *hns* promoter hexamers (−35 and −10), Transcription Start Site (TSS) region including the Discriminator, Shine-Dalgarno Sequence (SD) and DNA sequence encoding for the first three amino acids of H-NS are shown (**A**). The *hns* nucleotide sequence (gray bars) at –10 promoter motif (**B**) and TSS region (**C**) was overlaid with base distribution histograms for ppGpp/DskA inhibited promoters as freely redrawn using data by Sanchez-Vazqueza et al. [11].

Recently, a large collection of (p)ppGpp directly regulated genes has been obtained thanks to an extensive in vitro transcription analysis carried out on more than 100 promoters (not including *hns*) whose in vivo expression was affected at least twofold by the modified nucleotide [11]. Although not being conclusive, given the limited number of genes tested, this study permitted the identification of some DNA sequence signatures characteristic of the three classes of promoters: inhibited, activated, and unaffected. Thus, the sequence of *hns* promoter was superimposed to the histograms of base distributions for promoters inhibited by (p)ppGpp as determined by Sanchez-Vazquez et al. [11]. This comparison, illustrated as bar graphs in Figure 6, includes the −10 promoter element (the TATA box) and the transcriptional start site (TSS) region encompassing the discriminator (from position −1 to position −6 and the initial transcribed sequence. Concerning the -10 consensus sequence, although three bases, the thymines at positions −12 and −7 and the adenine at position −11, are poorly informative due to the high sequence conservation of TATA box in all promoters, *hns* shows an adenine at position −8 that was found to interact with RNAP [59] and is strongly favored in promoters negatively affected by (p)ppGpp (Figure 6B). Furthermore, the cytosine at position −13 (extended −10 region) and the spacer length of 17 bp are compatible with promoters repressed by (p)ppGpp (Figure 6A). The discriminator region with its high G+C content is a potential disadvantage to strand-separation at transcription initiation and has been long related with (p)ppGpp down-regulation [60]. Relatively to *hns* sequence, three cytosines occupy positions −4, −3 and −1, respectively, thus contributing to constitute a robust discriminator (Figure 6C). Notably, the cytosine at position −1 was found at very high frequency (~85%) in inhibited promoters since its presence is required to stabilize the incoming rNTP. Conservation of C-1 is crucial given that it reduces the abortive products formation and accelerates the RNAP escape from those promoters as the ones negatively controlled by (p)ppGpp that show short-lived open complexes [61,62]. According to these findings, band shift assays clearly indicate that the stability and/or formation of *hns* promoter-RNAP complexes is impaired by the presence of the modified nucleotide (Figure 5), given an explanation for the (p)ppGpp-dependent reduction in the in vitro transcriptional activity of *hns* promoter (Figure 4). Furthermore, the *hns* sequence analysis reveals that the initial rNTPs incorporated into *hns* mRNA (Figure 6C, positions A+2, C+3 and A+6) well match with transcripts repressed by this alarmone. Regarding the −35 consensus hexamer, no significant sequence difference has been observed between activated and inhibited promoters suggesting that this element does not actively participate to (p)ppGpp regulation [11]. Summarizing, the *hns* promoter exhibits the primary sequence determinants accounting for a direct regulation by (p)ppGpp as validated through in vitro experiments (Figure 4 and Figure 5).

In addition to (p)ppGpp, a second effector, playing a crucial role during the stringent response, is the transcription factor DksA. This small protein (17 kDa) binds RNAP through the secondary channel [63] and largely contributes to create, at protein–protein contact region, the (p)ppGpp binding site 2 [10]. Differently, site 1 is located on RNAP at the interface between β’ and ω subunits and does not need additional proteins to be formed [9]. DksA was found to function in synergy with (p)ppGpp strengthening its effects, either positive or negative, on transcription. Thus, DksA and (p)ppGpp together work better than either alone and up- or down-regulation depends on the intrinsic kinetic properties of the distinctive promoter controlled by (p)ppGpp [7,64,65,66,67]. In this context, there is no evidence that the preparations of RNAP, both the commercial and purified one in our laboratory used in in vitro assays (Figure 4 and Figure 5), contain appreciable amounts of DksA, and as a consequence these enzymes lack the (p)ppGpp binding site 2. Importantly, site 2 was found to greatly affect RNAP activity, more than site 1, particularly at inhibited promoters [10]. Thus, it is reasonable to assume that the (p)ppGpp-dependent effect, observed in the in vitro transcription assays (Figure 4), results from the occupancy of the only site 1, suggesting that repression of *hns* promoter activity might be underestimated and/or taking place at lower (p)ppGpp concentration in the presence of DksA as previously described for rRNA genes [65]. Ultimately, the (p)ppGpp-DksA synergism, better and promptly than (p)ppGpp alone, could fine tune the *hns* transcription rate in response to small changes of intracellular levels of p(p)ppGpp as observed in vivo using drugs addition, *relA/spoT* mutants and RelA over-expressing systems (Figure 1, Figure 2 and Figure 3). The molecular dissection of (p)ppGpp-DksA interplay might be the topic for a future study.
